# Polyvictimization and Cognitive Function in an Ethnic Minority Aging Population

**DOI:** 10.1093/geroni/igab046.1651

**Published:** 2021-12-17

**Authors:** Mengting Li, XinQi Dong

**Affiliations:** 1 Rutgers, The State University of New Jersey, New Brunswick, New Jersey, United States; 2 Rutgers University, Rutgers Institute for Health, New Jersey, United States

## Abstract

Globally, around 1 in 6 older adults experienced some form of elder mistreatment in community settings. However, little is known about the prevalence of polyvictimization, or experience of multiple forms of abuse, which may exacerbate negative outcomes over that of any one form of victimization in isolation. Data were drawn from the PINE study. Polyvictimization was defined as exposure to multiple forms of victimization, including psychological, physical, and sexual mistreatment, financial exploitation, and caregiver neglect. Cognitive function was evaluated by global cognition, episodic memory, executive function, working memory, and MMSE. Regression analyses were performed. Among 3153 participants, 128 experienced two forms of abuse while 12 experienced three or more forms of abuse. Polyvictimization was associated with lower global cognition (b=-0.05, SE=0.02, p<.05), episodic memory (b=-0.06, SE=0.03, p<.05), working memory (b=-0.14, SE=0.07, p<.05), and processing speed (b=-0.68, SE=0.33, p<.05). Interventions could target older adults with polyvictimization and protect their cognitive function.

